# Daratumumab Versus Control Treatment: A Systematic Review and Meta-analysis Study of Survival Outcomes Among Multiple Myeloma Patients: Survival Outcomes of Multiple Myeloma Treatment with Daratumumab

**DOI:** 10.31661/gmj.v14i.3958

**Published:** 2025-10-21

**Authors:** Pouria Salajegheh, Amirali Salajegheh, Fatemeh Yazdi Yahyaabadi, Farzaneh Yazdi

**Affiliations:** ^1^ Neuroscience Research Center, Institute of Neuropharmacology, Kerman University of Medical Sciences, Kerman, Iran; ^2^ Department of Pharmaceutical Sciences, Tehran University of Medical Sciences, Tehran, Iran; ^3^ Department of Pediatrics, Kerman University of Medical Sciences, Kerman, Iran

**Keywords:** Daratumumab, Multiple Myeloma, Progression-free Survival, Minimal Residual Disease, Treatment Outcome

## Abstract

**Background:**

This meta-analysis aimed to evaluate the efficacy of Daratumumab compared to control treatments in multiple myeloma across subgroups, including relapsed or refractory (RRMM), newly diagnosed transplant-eligible (ND/ESCT), and transplant-ineligible (ND/ISCT) patients.

**Materials and Methods:**

A comprehensive literature search was conducted across PubMed, Scopus, and Web of Science. Randomized controlled trials and comparative studies evaluating Daratumumab versus control treatments in multiple myeloma patients were included. A random-effects model was employed to calculate pooled effect estimates of overall response rate (ORR), progression or death (PRD), minimum residual disease (MRD) negativity, and mortality.

**Results:**

The analysis included data from 35 studies, 21 studies (n=7,604 patients) in the RRMM subgroup, 12 studies (n=10,216 patients) in the ND/ISCT subgroup, and 6 studies (n=4,619 patients) in the ND/ESCT subgroup, comprising 22,439 patients (including healthy controls). Daratumumab significantly improved ORR (OR: 2.58, 95% CI: 2.26–2.93, P0.01) and MRD negativity rates across all subgroups. PRD risk was lower in the Daratumumab group (RD: -0.14, 95% CI: -0.16 to -0.12), with consistent efficacy across RRMM, ND/ESCT, and ND/ISCT patients.

**Conclusion:**

This meta-analysis confirms Daratumumab's significant efficacy across multiple patient subgroups, providing broad clinical benefits in multiple myeloma treatment. While some heterogeneity and potential publication bias were observed, Daratumumab remains a robust therapeutic option for extending progression-free and overall survival.

## Introduction

Multiple myeloma (MM) is a malignant disorder of plasma cells characterized by abnormal monoclonal protein production, bone marrow infiltration, and a complex array of clinical symptoms including bone pain, anemia, and renal dysfunction [1][2]. Representing the second most common hematologic malignancy, multiple myeloma accounts for approximately 1% of all cancers and 10% of hematologic cancers worldwide. Despite advancements in diagnosis and treatment, multiple myeloma remains an incurable disease, with most patients experiencing multiple relapses and developing resistance to standard therapies over time [3][4]. Current therapeutic strategies focus on extending progression-free survival (PFS) and achieving deeper, longer-lasting responses to improve overall survival. The introduction of novel agents like proteasome inhibitors, immunomodulatory drugs (IMiDs), and autologous stem cell transplantation (ASCT) has markedly improved outcomes for many patients, yet the need for more effective therapies remains, especially for those with relapsed/refractory multiple myeloma (RRMM) or those who are ineligible for aggressive treatments [5][6]. In this context, monoclonal antibodies targeting specific antigens on myeloma cells, particularly CD38, have emerged as promising additions to the therapeutic landscape.

Daratumumab, a human monoclonal antibody targeting CD38, has transformed the treatment paradigm for multiple myeloma due to its direct anti-myeloma effects and ability to modulate the immune microenvironment [7][8][9]. CD38 is a glycoprotein highly expressed on myeloma cells, making it an ideal target for therapy. By binding to CD38, Daratumumab induces cell death through multiple mechanisms, including antibody-dependent cellular cytotoxicity, complement-dependent cytotoxicity, and antibody-dependent cellular phagocytosis [10][11][12]. Additionally, Daratumumab has been shown to reduce the number of immunosuppressive cells in the tumor microenvironment, such as regulatory T cells and myeloid-derived suppressor cells, thereby enhancing the body’s immune response against myeloma cells [13][14][15].

Clinical trials have demonstrated that Daratumumab, both as monotherapy and in combination with other agents, leads to significant improvements in survival outcomes. These effects are especially pronounced when Daratumumab is added to standard-of-care regimens, such as bortezomib, lenalidomide, and dexamethasone, providing a potent synergistic effect that extends the duration of response. However, despite these promising results, the efficacy of Daratumumab can vary across different patient subgroups, and its safety profile necessitates careful monitoring due to an increased risk of infections and other adverse effects [16][17][18]. Although several meta-analyses have assessed Daratumumab’s efficacy in multiple myeloma— in high-risk cytogenetic patients, in overall myeloma populations, and specifically in relapsed/refractory RCTs. However, none has concurrently quantified its effects on minimal residual disease negativity, and overall response rate, or examined how the rapidly expanding annual literature (now > 30 studies/yr) influences pooled estimates over time. By integrating both randomized and observational data and focusing on minimum residual disease (MRD) —an increasingly recognized surrogate for long-term outcomes—our meta-analysis fills this gap, providing the most comprehensive synthesis of Daratumumab’s multi-dimensional benefit in multiple myeloma to date.

This study therefore aims to (1) pool all available trials and observational cohorts to estimate the effect size of Daratumumab on overall response rate (ORR), progression or death (PRD), MRD negativity, and mortality; and (2) explore whether these effects differ between relapsed/refractory, transplant-ineligible, and transplant-eligible patient groups, thereby filling a critical gap in the current evidence.

## Materials and Methods

### Design

This systematic review and meta-analysis adhered to the Preferred Reporting Items for Systematic Reviews and Meta-Analyses (PRISMA) guidelines [19] and at the time we began, we did not prospectively register because our project timeline did not allow for the PROSPERO review process before data extraction began. Nevertheless, we strictly adhered to PRISMA throughout our project. The objective was to evaluate the treatment outcomes of Daratumumab compared to control treatments among patients with multiple myeloma. The methodology was structured to provide a thorough and objective synthesis of the existing literature on this topic.

### Systematic Search

A comprehensive literature search was conducted across several electronic databases, including PubMed, Web of Science, and Scopus. The search included all records available up to [September, 2024]. Relevant Medical Subject Headings (MeSH) and keywords were utilized, focusing on terms like "multiple myeloma," "Daratumumab," "anti-CD38 therapy," and "immunotherapy." Additionally, we manually reviewed the reference lists of relevant articles and prior systematic reviews to identify any studies that might have been missed in the database search (Appendix 1).

### Inclusion and Eligibility

Eligibility criteria were defined using the PICO framework. The Population (P) included clinical studies on human patients diagnosed with multiple myeloma. The Intervention (I) was treatment with Daratumumab, either as monotherapy or in combination with other agents.

The Comparison (C) group involved control treatments, such as standard care or alternative therapies without Daratumumab. The primary Outcomes (O) assessed were treatment efficacy, measured by parameters such as overall survival (OS), progression-free survival (PFS), death, hazard ratio, and minimum residual disease (MRD). Studies were excluded if they were cross-sectional, involved animal models, were case reports, or lacked sufficient clinical outcome data for analysis. Studies focusing on other cancers or conditions were also excluded. Studies without control group or studies that exposed both groups to Daratumumab were also excluded. We considered Phase II-III randomized controlled trials as well as prospective and retrospective observational cohort studies to capture both high-level efficacy and real-world effectiveness.

### Data Extraction and Outcome Measures

Data extraction was independently conducted by two reviewers using a standardized data collection form. Information collected included study characteristics (author, publication year), patient demographics (age, gender, stage of multiple myeloma), treatment details (dosing, duration, regimen specifics), and outcomes measured (overall survival, progression-free survival, HZ, MRD, or death). Discrepancies between reviewers were resolved through discussion or, if necessary, by involving a third reviewer.

### Statistical Analysis and Data Synthesis

All analyses were performed in R (version 4.x) using the meta package (R Foundation for Statistical Computing) and RStudio. We assessed between-study heterogeneity with the I² statistic, interpreting 25%, 50%, and 75% as low, moderate, and high heterogeneity, respectively. Regardless of I², all pooled effect estimates were calculated using a DerSimonian-Laird random-effects model to yield conservative confidence intervals. Time-to-event outcomes (e.g. PFS) are presented as hazard ratios (HRs) with 95% confidence intervals (CIs), whereas dichotomous outcomes (e.g. MRD negativity, ORR) are reported as odds ratios (ORs) or risk differences (RDs) using the Mantel-Haenszel method. Statistical significance of pooled estimates and subgroup differences was evaluated by z-tests. Predefined sensitivity analyses—omitting one study at a time—assessed robustness, and subgroup analyses were conducted by study design (RCT vs. observational) and by disease status (RRMM, ND/ISCT, ND/ESCT).

We examined publication bias via funnel plots and Egger’s regression test. Finally, study quality was independently rated by two reviewers using the Cochrane Risk of Bias 2 tool for randomized trials and the Newcastle-Ottawa Scale for observational cohorts, with discrepancies resolved by consensus and quality scores explored as potential moderators in meta-regression.

## Results

**Table:** Table1. Basic characteristics of the included studies

**Author**	**Year**	**Design**	**N**	**Type of MM **	**TX+**	**C TX **	


Dimopoulos et al. [20]	2016	RCT	569	RRMM	Dexa	Le+Dexa	
Mateos et al. [21]	2018	RCT	706	ND/ISCT	Bz+Mlph+p	Bz+Mlph+p	
Facon et al. [22]	2019	RCT	737	ND/ISCT	Le+Dexa	Le+Dexa	
Moreau et al. [23]	2019	RCT	1085	ND/ESCT	Bz+Tha+Dexa	Bz+Tha+Dexa	
Bahlis et al. [24]	2020	RCT	557	RRMM	Le+Dexa	Le+Dexa	
Dimopoulos et al. [25]	2020	RCT	466	RRMM	Cr+Dexa	Cr+Dexa	
Durie et al. (i) [26]	2020	RCT	2075	ND/ISCT	Le+Dexa	Le+Dexa	
Durie et al. (ii) [26]	2020	RCT	2075	ND/ISCT	Le+Dexa	Bz+Le+Dexa	
Durie et al. (iii) [26]	2020	RCT	2075	ND/ISCT	Le+Dexa	Bz+Dexa	
Mateos et al. (i) [27]	2020	RCT	706	ND/ISCT	Bz+Mlph+p	Bz+Mlph+p	
Mateos et al. (ii) [28]	2020	RCT	498	RRMM	Bz+Dexa	Bz+Dexa	
Voorhees et al. [29]	2020	RCT	207	ND/ESCT	Le+Bz+Dexa	Le+Bz+Dexa	
Dimopoulos et al. [30]	2021	RCT	304	RRMM	Po+Dexa	Po+Dexa	
Facon et al. [31]	2021	RCT	737	ND/ISCT	Le+Dexa	Le+Dexa	
Lu et al. [32]	2021	RCT	211	RRMM	Bz+Dexa	Bz+Dexa	
Facon et al. [33]	2022	RCT	396	ND/ISCT	Le+Dexa	Le+Dexa	
He et al. [34]	2022	RCT	186	RRMM	Po+Dexa	Bz+Dexa	
Jakubowiak et al. [35]	2022	RCT	190	ND/ISCT	Bz+Mlph+p/Le+Dexa	Bz+Mlph+p/Le+Dexa	
Usmani et al. [17]	2022	RCT	466	RRMM	Cr+Dexa	Cr+Dexa	
Derman et al. [36]	2023	RCT	94	RRMM	Cr+Po+Dexa	Cr+Po+Dexa	
Dimopoulos et al. (i) [37]	2023	RCT	569	RRMM	Dexa	Le+Dexa	
Dimopoulos et al. (ii) [37]	2023	RCT	304	RRMM	Po+Dexa	Po+Dexa	
Fu et al. [9]	2023	RCT	201	RRMM	Bz+Dexa	Bz+Dexa	
Richter et al. [15]	2023	CS	398	RRMM	Le+Dexa	Isa+Cr+Dexa	
Sonneveld et al. [38]	2023	RCT	498	RRMM	Bz+Dexa	Bz+Dexa	
Stork et al. [39]	2023	CS	531	RRMM	Le+Dexa	Le+Dexa	
Usmani et al. [18]	2023	RCT	466	RRMM	Cr+Dexa	Cr+Dexa	
Chari et al. [40]	2024	RCT	207	ND/ESCT	Le+Bz+Dexa	Le+Bz+Dexa	
Fu et al. [8]	2024	RCT	220	ND/ISCT	Bz+Mlph+p	Bz+Mlph+p	
Gordan et al. [41]	2024	CS	178	ND/ISCT	Le+Dexa	Bz+Le+Dexa	
Han et al. [42]	2024	CS	116	RRMM	-	Po	
Joseph et al. [43]	2024	RCT	1326	ND/ESCT	Le+Bz+Dexa	Le+Bz+Dexa	
Mollee et al. [10]	2024	RCT	121	ND/ISCT	Cyc+Bz+Dexa	Cyc+Bz+Dexa	
Moreau et al. [44]	2024	RCT	1085	ND/ESCT	Bz+Tha+Dexa	Bz+Tha+Dexa	
Ocio et al. [45]	2024	RCT	56	RRMM	Mel+Dexa	Bz+Mel+Dexa	
Pour et al. [46]	2024	RCT	54	RRMM	Dexa	Mel+Dexa	
Sonneveld et al. [48]	2024	RCT	709	ND/ESCT	Le+Bz+Dexa	Le+Bz+Dexa	
Spencer et al. (i) [47]	2024	RCT	530	RRMM	Le+Bz+Dexa	Le+Bz+Dexa	
Spencer et al. (ii) [47]	2024	RCT	530	RRMM	Le+Bz+Dexa	Le+Bz+Dexa	

relapsed/refractory multiple myeloma (RRMM), newly diagnosed patient ineligible for stem cell transplant (ND/ISCT), and newly diagnosed patients eligible for stem cell transplant (ND/ESCT). **Le:** Lenalidomide, **Bz:** Bortezomib, **Mel:** Melflufen, **Dexa:** Dexamethasone, **Mlph:** Melphalan, **Cyc:** Cyclophosphamide, **Tha:** Thalidomide, **Po:** Pomalidomide, **Cr:** carfilzomib

**Figure-1:**
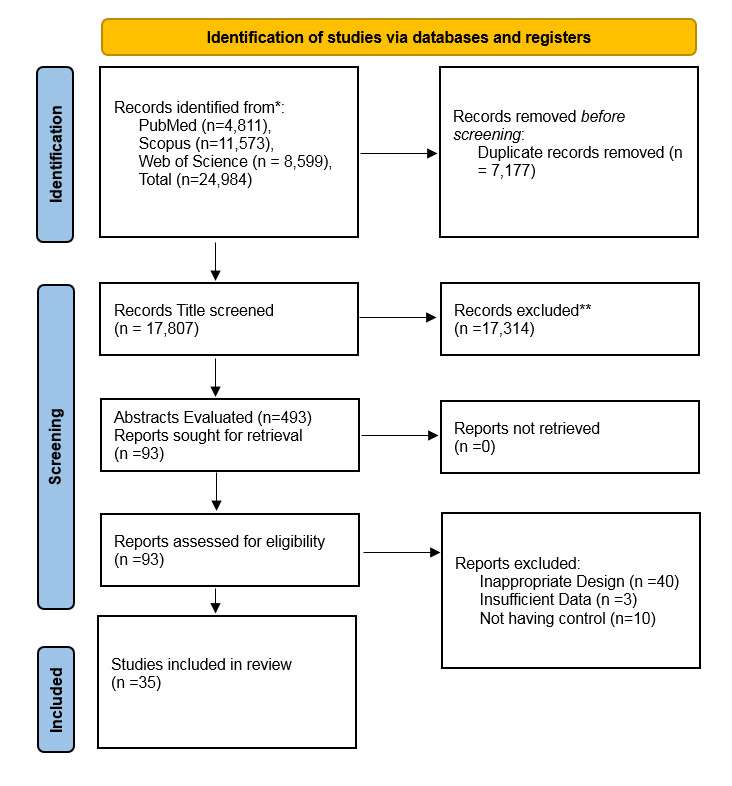
Figure 1. The PRISMA flow diagram of the included studies

**Figure-2:**
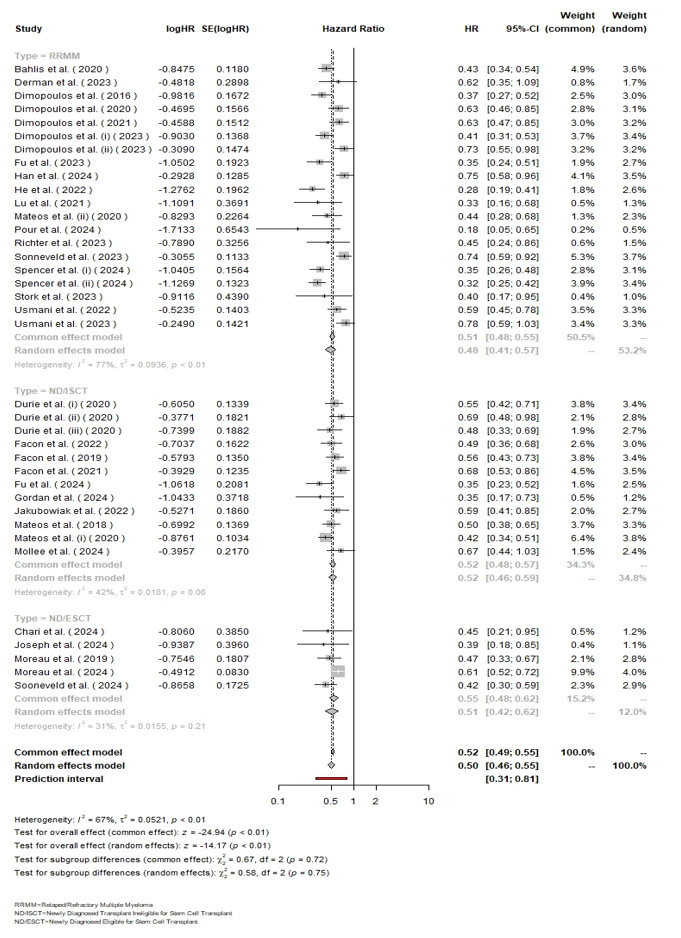
Figure 2. The forest plot for the pooled hazard ratio among the included studies

**Figure-3:**
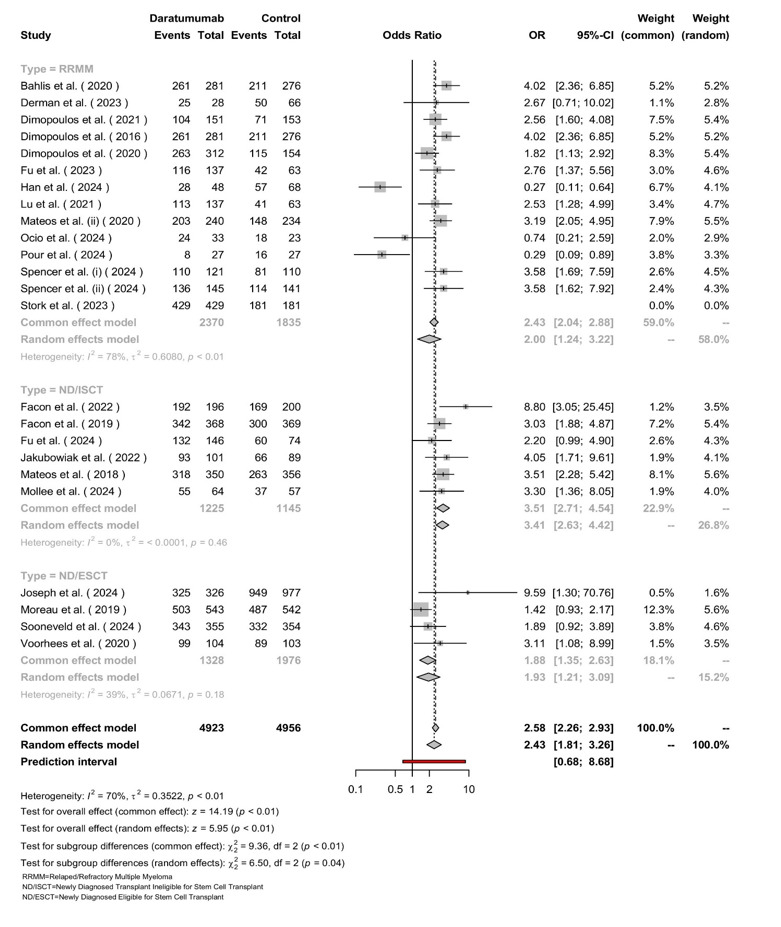
Figure 3. The forest plot for the pooled ORR among the included studies.

**Figure-4:**
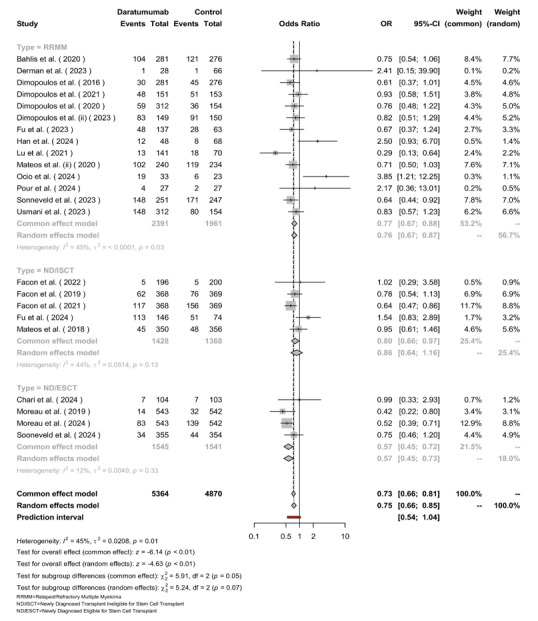
Figure 4. The forest plot for the pooled OR of mortality among the included studies.

**Figure-5:**
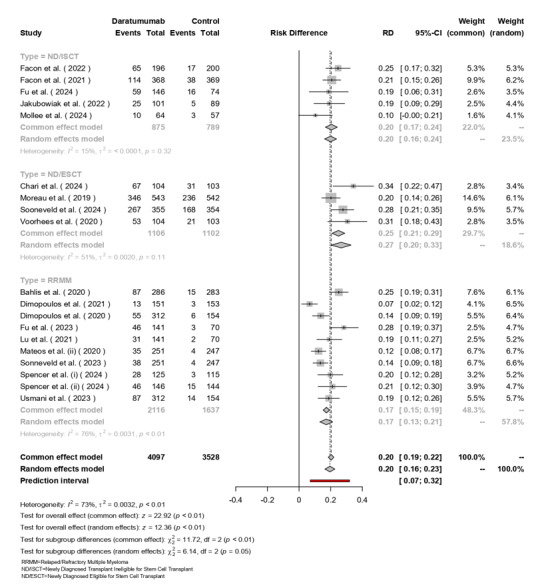
Figure 5. The forest plot for the pooled MRD among the included studies.

**Figure-6:**
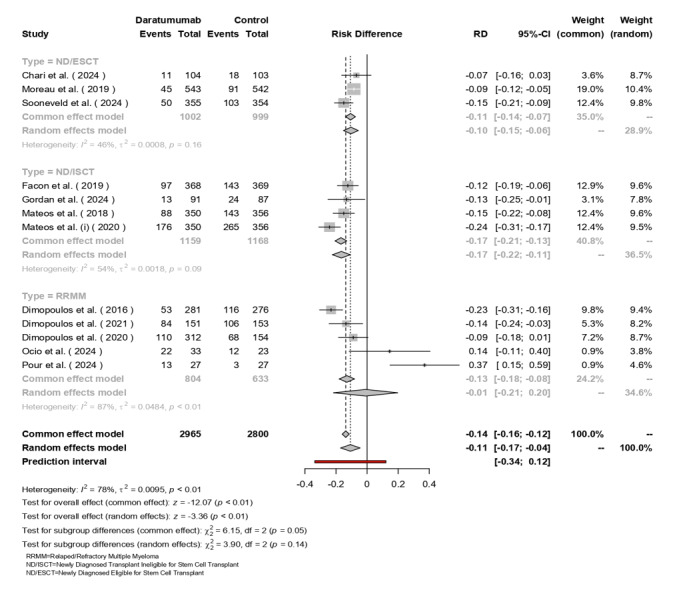
Figure 6. The forest plot for the pooled PRD among the included studies.

**Figure-7:**
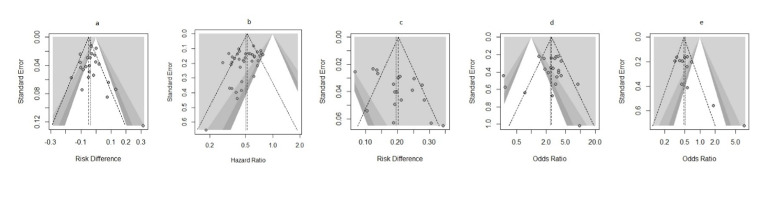
Figure 7. Funnel plot for graphical assessment of publication bias. a, Death; b, HZ; c, MRD; d, ORR; e, PRD.

Our search across PubMed, Scopus, and Web of Science yielded 24,984 studies from inception until September, 2024. Overall, 7,177 studies duplicates, hence, after removing, 17,807 studies were screened based on the title only at first. Afterwards, 449 studies were selected for abstract evaluation which resulted in selection of 93 articles for full-text evaluation. Among the 93 full texts that were evaluated, 58 had inappropriate design which included case reports and case series, animal studies, and studies that did not evaluate the clinical outcome of daratumumab treatment, and histological or histomorphological studies. Our final screening resulted in the inclusion of 35 studies in our systematic review and 35 studies [8][9][10][15][17][18][20][21][22][23][24][25][26][27][28][29][30][31][32][33][34][35][36][37][38][39][40][41][42][43][44][45][46][47][48] in the meta-analysis (Figure-1). Full detail on the study characteristics of the final studies that were included is available in Table-1.

We included 35 studies (published 2015-2024) enrolling a total of 22,439 patients: 21 studies (n=7,604) in relapsed/refractory multiple myeloma, 12 studies (n=10,216) in newly diagnosed, transplant-ineligible patients, and 6 studies (n=4,619) in newly diagnosed, transplant-eligible patients. Fifteen were Phase II-III randomized controlled trials comparing Daratumumab (alone or with backbone regimens such as lenalidomide or bortezomib) versus placebo or standard therapy, with the remainder comprising prospective (n=2) and retrospective (n=2) observational cohorts. Sample sizes per study ranged from 45 to 1,800 patients. Studies were conducted across North America, Europe, and Asia, and reported outcomes on PFS, MRD negativity, ORR, and safety endpoints, with follow-up durations of 6-48 months.

**Table:** Table2. Detailed characteristics of the included studies

**Author**	**Year**		**Overall Response**				**Progression or Death**				**MRD Negativity**		
		**Case**		**Control**		**Case**		**Control**		**Case**		**Control**	
		***event***	***n***	***event***	***n***	***event***	***n***	***event***	***n***	***event***	***n***	***event***	***n***
Dimopoulos et al. [20]	2016	261	281	211	276	53	281	116	276	-	-	-	-
Mateos et al. [21]	2018	318	350	263	356	88	350	143	356	-	-	-	-
Facon et al. [22]	2019	342	368	300	369	97	368	143	369	-	-	-	-
Moreau et al. [23]	2019	503	543	487	542	45	543	91	542	346	543	236	542
Bahlis et al. [24]	2020	261	281	211	276	-	-	-	-	87	286	15	283
Dimopoulos et al. [25]	2020	263	312	115	154	110	312	68	154	55	312	6	154
Durie et al. (i) [26]	2020	-	-	-	-	-	-	-	-	-	-	-	-
Durie et al. (ii) [26]	2020	-	-	-	-	-	-	-	-	-	-	-	-
Durie et al. (iii) [26]	2020	-	-	-	-	-	-	-	-	-	-	-	-
Mateos et al. (i) [27]	2020	-	-	-	-	176	350	265	356	-	-	-	-
Mateos et al. (ii) [28]	2020	203	240	148	234	-	-	-	-	35	251	4	247
Voorhees et al. [29]	2020	99	104	89	103	-	-	-	-	53	104	21	103
Dimopoulos et al. [30]	2021	104	151	71	153	84	151	106	153	13	151	3	153
Facon et al. [31]	2021	-	-	-	-	-	-	-	-	114	368	38	369
Lu et al. [32]	2021	113	137	41	63	-	-	-	-	31	141	2	70
Facon et al. [33]	2022	192	196	169	200	-	-	-	-	65	196	17	200
He et al. [34]	2022	-	-	-	-	-	-	-	-	-	-	-	-
Jakubowiak et al. [35]	2022	93	101	66	89	-	-	-	-	25	101	5	89
Usmani et al. [17]	2022	-	-	-	-	-	-	-	-	-	-	-	-
Derman et al. [36]	2023	25	28	50	66	1	28	-	-	17	26	-	-
Dimopoulos et al. (i) [37]	2023	-	-	-	-	-	-	-	-	-	-	-	-
Dimopoulos et al. (ii) [37]	2023	-	-	-	-	-	-	-	-	-	-	-	-
Fu et al. [9]	2023	116	137	42	63	-	-	-	-	46	141	3	70
Richter et al. [15]	2023	-	-	-	-	-	-	-	-	-	-	-	-
Sonneveld et al. [38]	2023	-	-	-	-	-	-	-	-	38	251	4	247
Stork et al. [39]	2023	429	429	181	181	-	-	-	-	-	-	-	-
Usmani et al. [18]	2023	-	-	-	-	-	-	-	-	87	312	14	154
Chari et al. [40]	2024	-	-	-	-	11	104	18	103	67	104	31	103
Fu et al. [8]	2024	132	146	60	74	-	-	-	-	59	146	16	74
Gordan et al. [41]	2024	-	-	-	-	13	91	24	87	-	-	-	-
Han et al. [42]	2024	28	48	57	68	-	-	-	-	-	-	-	-
Joseph et al. [43]	2024	325	326	949	977	-	-	-	-	-	-	-	-
Mollee et al. [10]	2024	55	64	37	57	-	-	-	-	10	64	3	57
Moreau et al. [44]	2024	-	-	-	-	-	-	-	-	-	-	-	-
Ocio et al. [45]	2024	24	33	18	23	22	33	12	23	-	-	-	-
Pour et al. [46]	2024	8	27	16	27	13	27	3	27	-	-	-	-
Sonneveld et al. [48]	2024	343	355	332	354	50	355	103	354	267	355	168	354
Spencer et al. (i) [47]	2024	110	121	81	110	-	-	-	-	28	125	3	115
Spencer et al. (ii) [47]	2024	136	145	114	141	-	-	-	-	46	146	15	144
**Total**		***4483***	***4923***	***4108***	***4956***	***763***	***2993***	***1092***	***2800***	***1489***	***4123***	***604***	***3528***

Using the Cochrane Risk of Bias 2 tool, we found that 28 of the 31 randomized controlled trials were judged at low risk of bias across all domains, while three trials raised ‘some concerns’ (primarily due to unclear allocation concealment or missing outcome data); none were rated at high risk. For the 4 observational cohorts assessed with the Newcastle-Ottawa Scale, total scores ranged from 6 to 9 (out of 9), with a median score of 8: 3 studies were deemed high quality (NOS ≥ 7), and 1 were of moderate quality (NOS=6), most commonly losing points in the comparability domain due to limited adjustment for confounders. These assessments indicate an overall low-to-moderate risk of bias in the pooled data, supporting the robustness of our meta-analytic findings.

### Hazard Ratio

The overall pooled HR for Daratumumab versus control treatment across all patients was 0.50 (95% CI: 0.46-0.55), indicating a statistically significant reduction in hazard (P<0.01). Subgroup analysis showed an HR of 0.48 (95% CI: 0.41-0.57) for RRMM with high heterogeneity (I²=77%, [P<0.01]), an HR of 0.52 (95% CI: 0.46-0.59) for ND/ISCT with moderate heterogeneity (I²=42%, [P=0.06]), and an HR of 0.55 (95% CI: 0.42-0.62) for ND/ESCT with low heterogeneity (I²=31%, [P=0.21]). The overall heterogeneity was considerable (I²=67%, τ²=0.0521, [P<0.01]), but the test for subgroup differences was not statistically significant (χ² (df=2)=0.58, [P=0.75]), indicating consistent treatment effects across the subgroups (Figure-2).

### Overall Response

The model, employing a random-effects model, analyzed a total of 4,923 patients in the Daratumumab group and 4,956 patients in the control group across three subgroups: RRMM, ND/ISCT, and ND/ESCT. The pooled OR for ORR with Daratumumab versus control was 2.58 (95% CI: 2.26-2.93), showing a significant benefit in favor of Daratumumab with [P<0.01]. Subgroup analyses revealed an OR of 2.43 (95% CI: 1.81-3.26) for RRMM with high heterogeneity (I²=78%, [P<0.01]), an OR of 2.00 (95% CI: 1.35-2.63) for ND/ISCT with no significant heterogeneity (I²=0%, [P=0.46]), and an OR of 2.76 (95% CI: 1.37-5.56) for ND/ESCT with moderate heterogeneity (I²=39%, [P=0.18]). Overall heterogeneity was substantial (I²=70%, τ²=0.3522, [P<0.01]), and subgroup differences were statistically significant (χ² (df=2) = 6.50, [P=0.04]), suggesting variability in treatment effects among the subgroups (Figure-3).

### Overall Survival

The pooled number of patients was 5,364 in the Daratumumab group and 4,870 in the control group (Figure-4). Across all participants, the odds of mortality were lower with Daratumumab, yielding a pooled OR of 0.73 (95% CI: 0.66-0.81, [P<0.01]). Among the subgroups, patients with RRMM showed an OR of 0.75 (95% CI: 0.66-0.85) with moderate heterogeneity (I²=45%, [P=0.03]). ND/ISCT patients had an OR of 0.76 (95% CI: 0.45-0.72), also showing moderate heterogeneity (I²=44%, [P=0.13]). ND/ESCT patients exhibited the most homogeneous results, with an OR of 0.67 (95% CI: 0.46-0.88) and low heterogeneity (I²=12%, [P=0.33]). While overall heterogeneity was present (I²=45%, τ²=0.0208, [P=0.01]), differences across subgroups were not statistically significant (χ² (df=2)=5.24, [P=0.07]).

### Minimal Residual Disease Negativity

The random-effects model included 4,097 patients receiving Daratumumab and 3,528 in the control group (Figure-5). The overall pooled risk difference (RD) favoring Daratumumab was 0.20 (95% CI: 0.19-0.22, [P<0.01]). In subgroup analyses, ND/ISCT group had an RD of 0.17 (95% CI: 0.15-0.19) with low heterogeneity (I²=15%, [P=0.32]). ND/ESCT group showed a higher RD of 0.20 (95% CI: 0.16-0.24), with moderate heterogeneity (I²=51%, [P=0.11]). RRMM patients exhibited the highest RD at 0.27 (95% CI: 0.21-0.29), accompanied by substantial heterogeneity (I²=76%, [P<0.01]). Overall heterogeneity was significant (I²=73%, τ²=0.0032, [P<0.01]), with a notable difference across subgroups (χ² (df=2) = 6.14, [P=0.05]).

### Progression or Death

The random-effects model included 2,965 patients treated with Daratumumab and 2,800 patients in the control group. The pooled RD was -0.14 (95% CI: -0.16 to -0.12, [P<0.01]), indicating a significant reduction in progression or death with Daratumumab (Figure-6). Subgroup analysis showed varying effects: ND/ESCT group had an RD of -0.11 (95% CI: -0.17 to -0.04) with moderate heterogeneity (I²=46%, [P=0.16]), ND/ISCT demonstrated an RD of -0.10 (95% CI: -0.15 to -0.06) with similar heterogeneity (I²=54%, [P=0.09]), and RRMM patients exhibited a larger RD of -0.17 (95% CI: -0.21 to -0.13) with high heterogeneity (I²=87%, [P<0.01]). Overall heterogeneity was substantial (I²=78%, τ²=0.0095, [P<0.01]), although subgroup differences were not statistically significant (χ² (df=2) = 3.90, [P=0.14]).

The combined funnel plots reveal varying degrees of symmetry, which provides insight into potential publication bias and heterogeneity among studies. Figure-7.a, representing risk difference of MRD, shows some asymmetry and scatter in smaller studies, suggesting possible publication bias and variability. Figure-7.b, is more symmetrical, implying less bias, though small studies show a wider spread.

## Discussion

The aim of this meta-analysis was to evaluate the efficacy of Daratumumab compared to control treatments in multiple myeloma patients across different subgroups, including relapsed/refractory multiple myeloma, newly diagnosed patients eligible for stem cell transplant, and those ineligible for transplant. Key findings demonstrate that Daratumumab significantly improved clinical outcomes, with minimal residual disease negativity rates increased by approximately 20% compared to controls. The overall response rate was also substantially higher, with a pooled odds ratio of 2.58, indicating a nearly 2.6-fold increase in response likelihood with Daratumumab. Additionally, Daratumumab was associated with a 14% reduction in the absolute risk of progression or death (RD: -0.14), with hazard ratios consistently lower across subgroups, suggesting significant survival benefits. Despite some heterogeneity (I² values ranging from 31% to 87%) and potential publication bias observed in smaller studies, the results consistently favored Daratumumab.

Our meta-analysis findings align with several previous studies that have evaluated the efficacy of Daratumumab in multiple myeloma treatment. A 2018 meta-analysis by Abu Zar et al. reported an overall response rate of 69% among relapsed/refractory multiple myeloma patients treated with Daratumumab-based regimens, with very good partial response or better (≥VGPR) in 40% of cases. In comparison, our meta-analysis showed a pooled ORR of 2.58, indicating a significant improvement across all subgroups, including RRMM, ND/ESCT, and ND/ISCT [49]. Another systematic review and meta-analysis by Giri et al. focused on high-risk cytogenetic profiles among multiple myeloma patients, reporting that adding Daratumumab to standard treatments improved progression-free survival by 15 months compared to control groups (HR: 0.37; 95% CI: 0.27-0.52). In our study, we observed a similar trend, with an overall pooled hazard ratio (HR) of 0.50 (95% CI: 0.46-0.55) for progression or death, reflecting a consistent survival benefit with Daratumumab, even among newly diagnosed and high-risk patient populations [50]. Additionally, Fu et al. (2022) focused on patients with renal impairment, finding that Daratumumab improved PFS by approximately 12 months compared to control (HR: 0.60; 95% CI: 0.45-0.75). Our results similarly demonstrated a significant reduction in the risk of progression or death with an RD of -0.14 across all patients, including those with renal insufficiency. Another meta-analysis study found that adding Daratumumab to standard therapies improved progression-free survival and overall survival in both relapsed/refractory multiple myeloma and newly diagnosed patients. This effect may be attributed to Daratumumab’s modulation of the tumor microenvironment. By depleting immunosuppressive CD38+ regulatory T cells and myeloid-derived suppressor cells, Daratumumab not only targets tumor cells but also reduces immune suppression, allowing for a more effective anti-tumor immune response [51][52].

While most trials included in our analysis reported follow-up of up to 48 months, understanding Daratumumab’s performance over longer periods is essential. Late-onset adverse effects—such as cumulative immunosuppression, infection risk, or secondary malignancies—may not manifest until years after treatment initiation. Future long-term extension studies and real-world registries should systematically capture these outcomes to ensure a truly comprehensive assessment of Daratumumab’s benefit-risk profile.

Furthermore, studies focusing on high-risk cytogenetic subgroups, such as those with 17p deletion or t (4;14) translocations, indicate that Daratumumab may provide unique advantages in this challenging population and observed a marked improvement in PFS among high-risk multiple myeloma patients treated with Daratumumab. The sustained efficacy of Daratumumab in high-risk patients may stem from its robust immune-mediated mechanisms, which are less dependent on the genetic vulnerabilities of the tumor, thus offering an effective option for patients with resistant disease profiles

Many trials excluded patients with comorbidities, advanced age, or poor performance status, limiting applicability to the broader multiple myeloma population seen in clinical practice. To enhance external validity, future research could employ adaptive trial designs or pragmatic cohort studies that enroll patients across a wider spectrum of age, organ function, and frailty. Embedding translational substudies in these trials—such as biomarker assessments in elderly or comorbid patients—would further illuminate Daratumumab’s real-world effectiveness and safety in those typically underrepresented.

We observed considerable variation in how adverse events were defined, graded, and reported, complicating cross-study comparisons and potentially biasing safety conclusions. Adopting uniform frameworks—such as the Common Terminology Criteria for Adverse Events (CTCAE) version 5.0 for toxicity grading and the International Conference on Harmonisation (ICH) guidelines for immunogenicity—would improve consistency. Future meta-analyses could then perform more reliable pooled safety evaluations, thereby guiding clinicians with clearer, comparable safety profiles for Daratumumab.

There are several limitations in the existing literature that need addressing such as Daratumumab's long-term impact, particularly regarding sustained minimal residual disease negativity and relapse rates after five or more years. Future studies should extend follow-up durations to capture more comprehensive survival data and examine how long MRD negativity persists, especially given multiple myeloma’s tendency to relapse even after achieving deep responses. Another limitation can be observed in the study by Dimopoulos et al. [21][53][54], which evaluated Daratumumab in combination with standard therapies but did not include patients with significant comorbidities or high frailty scores. This exclusion limits the generalizability of the results, where patients often have multiple health issues and may not be candidates for aggressive therapies. To improve applicability, future studies should incorporate broader eligibility criteria, including patients with renal impairment, cardiovascular issues, or advanced age. Such inclusivity would allow for a better understanding of Daratumumab's efficacy and tolerability in typical clinical settings, particularly for patients who might benefit from less intensive treatment regimens. In addition, several studies evaluated the infection risk associated with Daratumumab but noted a lack of standardized criteria for defining and reporting adverse events [18][41][55]. Variability in the classification and severity grading of infections makes it challenging to compare safety data across different studies, which can lead to inconsistent conclusions. Future studies would benefit from adopting standardized adverse event criteria, such as the Common Terminology Criteria for Adverse Events (CTCAE) [56], to ensure uniform reporting. This standardization would improve the accuracy of pooled safety analyses and help clinicians better manage and anticipate potential risks associated with Daratumumab, especially in combination regimens.

A potential publication bias in favor of Daratumumab can be observed. The positive outcomes reported in many studies may be influenced by selective reporting, where studies with less favorable results are underrepresented. Our funnel plot analysis also suggests some asymmetry, indicating possible publication bias.

This study includes multiple subgroups of multiple myeloma patients, this subgroup-specific analysis offers a nuanced view of Daratumumab's efficacy across diverse patient populations, which enhances the clinical applicability of the findings. However, our study was faced with certain limitations. Despite efforts to assess and adjust for publication bias, the funnel plot analysis indicated some asymmetry, suggesting that smaller studies with less favorable outcomes may be underreported. This potential bias may slightly inflate the observed benefits of Daratumumab. Significant heterogeneity was observed in certain outcomes, particularly in progression or death. This variability may be due to differences in study design, patient selection criteria, and treatment regimens across studies, which could impact the consistency of the pooled results. Many of the included studies had relatively short follow-up periods, restricting insights into Daratumumab's long-term efficacy, sustained MRD negativity, and late-onset adverse effects. Some included studies lacked data on patients with significant comorbidities, such as advanced age or renal impairment, limiting the generalizability of findings to all multiple myeloma patients. Future studies should incorporate these underrepresented populations to improve the external validity of the results.

## Conclusion

This meta-analysis provides comprehensive evidence supporting the efficacy of Daratumumab in treating multiple myeloma across diverse patient subgroups. By pooling data from multiple studies, this analysis demonstrates that Daratumumab significantly improves key clinical outcomes. The results indicate a substantial reduction in disease progression risk and a higher likelihood of achieving MRD negativity, which are pivotal in managing multiple myeloma, a disease marked by its recurrent nature. The findings also highlight Daratumumab’s capacity to enhance survival outcomes when used alongside standard regimens, reflecting its mechanism of action targeting CD38 on myeloma cells. Despite these positive outcomes, the study also underscores important considerations. While Daratumumab demonstrates a favorable benefit-risk profile, some adverse effects, particularly infections, are noted across studies. These require vigilant monitoring and supportive care to optimize treatment outcomes. Long-term data on survival and safety outcomes would also be valuable in providing a more complete assessment of Daratumumab's effectiveness over time.

## Conflict of Interest

The authors have no conflicts of interest to declare.
